# Dosimetric evaluation of 120-leaf multileaf collimator in a Varian linear accelerator with 6-MV and 18-MV photon beams

**DOI:** 10.4103/0971-6203.42757

**Published:** 2008

**Authors:** R. Mohan, K. Jayesh, R. C. Joshi, Maha Al-idrisi, P. Narayanamurthy, Saroj Kumar Das Majumdar

**Affiliations:** Department of Radiation Oncology, King Fahad Specialist Hospital, Ministry of Health, Dammam, Kingdom of Saudi Arabia; 1 Department of Physics, Acharya Nagarjuna University, Guntur, Andhra Pradesh, India

**Keywords:** Dose rate, multileaf collimators, surface dose, three-dimensional conformal radiotherapy and intensity-modulated radiotherapy

## Abstract

In this study the dosimetric characteristics of 120-leaf multileaf collimators (MLCs) were evaluated for 6-MV and 18-MV photon beams. The dose rate, percentage depth dose, surface dose, dose in the build-up region, beam profile, flatness, symmetry, and penumbra width were measured using three field-defining methods: (i) ‘Jaw only’, (ii) ‘MLC only’, and (iii) ‘MLC+Jaw’. Analysis of dose rate shows that the dose rate for ‘MLC only’ field was higher than that for ‘Jaw only” and ‘MLC+Jaw’ fields in both the energies. The ‘percentage of difference’ of dose rates between ‘MLC only’ and ‘MLC+Jaw’ was (0.9% to 4.4%) and (1.14% to 7%) for 6 MV and 18 MV respectively. The surface dose and dose in the build-up region were more pronounced for ‘MLC only’ fields for both energies, and no significant difference was found in percentage depth dose beyond dmax for both energies. Beam profiles show that flatness and symmetry for both the energies were less than the 3%. The penumbra width for ‘MLC only’ field was more than that for the other two field-defining methods by (1 to 2 mm) and (0.8 to 1.3 mm) for 6-MV and 18-MV photon beams respectively. Analysis of ‘width of 50% dose level’ of the beam profiles at dmax to reflect the field size shows 1 to 2 mm more for 6-MV photons and 2.2 to 2.4 mm morefor 18-MV photons for ‘MLC only’ fields. The results of this study suggest that the characteristics of 120-leaf MLC system with 6 MV and 18 MV are same in all aspects except the surface dose, penumbra, dose in the build-up region, and width of 50% dose levels.

## Introduction

The three-dimensional conformal radiotherapy (3D-CRT), intensity-modulated radiotherapy (IMRT), and image-guided radiotherapy (IGRT) are the most advanced techniques in radiotherapy, which use irregular fields–using multileaf collimators in a linear accelerator. The accuracy of these techniques depends on dosimetric characteristics of the multileaf collimators. There is an option for optimizing the jaws to the irregular MLC field to reduce the scattered radiation and intra- and inter-leaf radiation leakage beyond the field. In this study, 120-leaf MLC system has been taken to compare and differentiate their characteristics with 6-MV and 18-MV photon beams.

The MLC system in Varian linear accelerator is used as a tertiary collimator, that is, below the collimator jaws. When both ‘X’ and ‘Y’ jaws are optimized to the MLC field, the surface dose decreases by reducing the intra- and inter-leaf leakage radiation.[1] The dosimetric characteristics include dose rates, percentage depth doses, surface dose, dose in the build-up region, penumbra, and width of 50% dose levels.

## Materials and Methods

Varian 2300CD CLINAC linear accelerator with 6-MV and 18-MV photon beams and 120-leaf multileaf collimator. Eclipse treatment planning system, PTW MP3 3D water phantom with 0.125-cc field and reference chambers, 0.6-cc farmer chamber, and PTW solid water (slab) phantom.

### A. 120-leaf MLC system

The Varian 120-leaf MLC system consists of an MLC head assembly and control system. The MLC system is attached to the head of the CLINAC 2300CD as a tertiary collimator consisting a pair of 60 opposed leaves. These leaves are mounted in two leaf banks below ‘X’ jaws. The leaf width at the isocenter for each of the central 80 leaves is 0.5 cm; and for all others, it is 1.0 cm.

### B. Measurement of dosimetric characteristics

Measurements were made in water using PTW MP3 water phantom using the following methods:

- MLC field with jaws optimized (MLC+Jaw) — optimization method
- MLC field with jaws parked at 35×35 cm (MLC only) — non-optimization method
- Jaw field with X1, X2, Y1, and Y2 jaws (Jaw only)

### C. Dose rate (DR)

The dose rate was measured in a PTW solid water (slab) phantom using a 0.6-cc PTW (PTW-Freiburg) waterproof ion chamber and a PTW UNIDOS digital electrometer. The solid phantom is tissue equivalent with a density of 1.045 g/cc; its dimensions are 30×30×30 cm (width×length×depth). It has a slot to position the 0.6-cc chamber. All measurements were made in source-to-surface distence(SSD) setup of 100 cm for field sizes 5×5 cm², 10×10 cm², 15×15 cm², 20×20 cm², and 30×30 cm² using IAEA TRS-398 absolute dose calculation protocol, for all the above-mentioned fields for 6-MV and 18-MV photon beams at a reference of depths 5 cm and 10 cm respectively.

### D. Percentage depth dose and beam profile

The central axis percentage depth dose (PDD) and beam profile were measured using PTW MP3 3D radiation field analyzer system controlled by Mephysto computer software. The RFA consists of a cubic water tank with inner dimensions 60×50×40.75 cm (width×length×depth). The drive mechanism of the scanning system has a positional accuracy of ±0.5 mm and reproducibility of ±0.1 mm. Semi flex cylindrical chambers, 0.125 cc, were used. The measuring chamber was positioned perpendicular to the radiation beam and parallel to the water surface. A reference detector was placed in one quadrant corner of the radiation field so that it does not interfere with the reading of the field detector. The PDD and beam profile were measured for source-to-surface (SSD) = 100 cm, for the square fields 5×5 cm², 10×10 cm², 20×20 cm² and 30×30 cm², using all the three field-defining methods as described in section B.

## Results and Discussion

**Figure 1:**
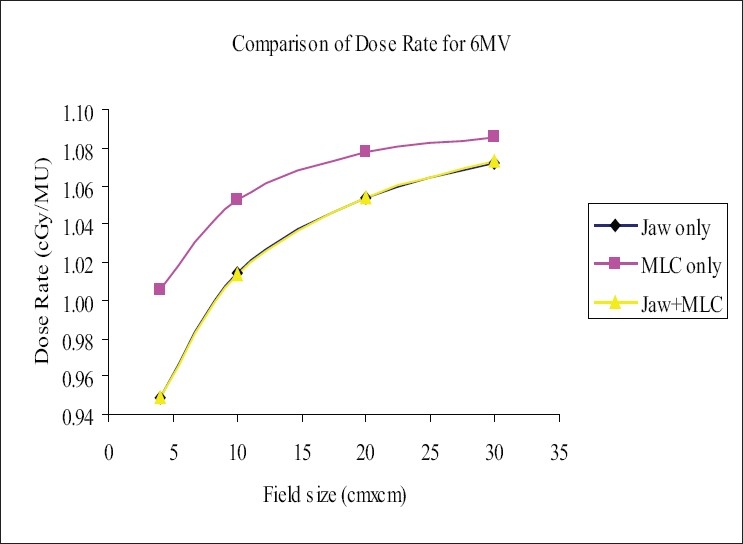
Comparison of dose rate (DR) for 6-MV photon beam, ‘Jaw only’ uses standard collimator jaws for field definition, ‘MLC only’ MLC field with jaws parked at 35×35 cm, non-optimization method, ‘MLC+Jaw’ MLC field with jaws optimized (zero gap), optimization method

**Figure 2:**
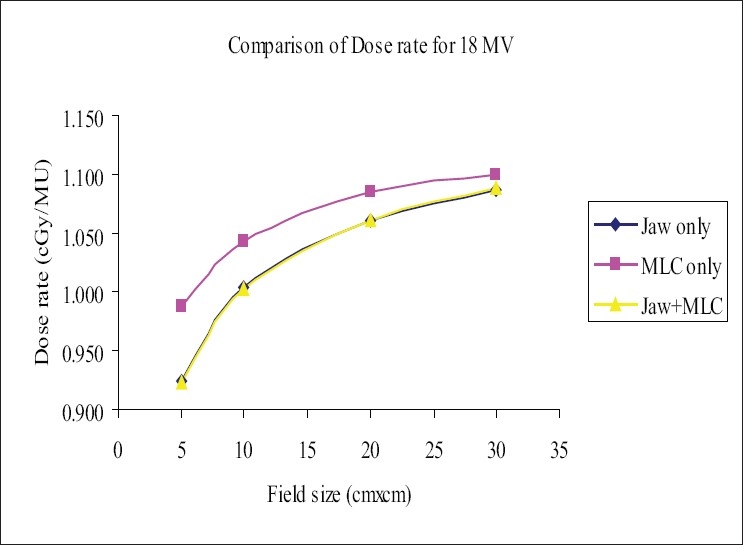
Comparison of dose rate (DR) for 18-MV photon beam, ‘Jaw only’ uses standard collimator jaws for field definition, ‘MLC only’ MLC field with jaws parked at 35×35 cm, non-optimization method, ‘MLC+Jaw’ MLC field with jaws optimized (zero gap), optimization method

Dose rate (DR) in medium was measured as described in section C, at a reference depth of 5 cm for 6-MV and 10 cm for 18-MV photon beams. The increases in the DR as shown in Figures 1 and 2 were more pronounced for the fields defined by ‘MLC only’ and had higher values compared to those for the fields defined by ‘Jaw only’ and/or ‘MLC+Jaw’. The range of ‘percentage of difference’ between the ‘MLC only’ field and ‘MLC+Jaw’ field was (0.9% to 4.40%) and (1.14% to 7%) for 6-MV and 18-MV photons respectively. It was found that there was no significant difference between the dose rates of ‘MLC+Jaw’ and ‘Jaw only” fields in both the energies. The increase in dose rate due to MLC+Jaw fields may be due to increase in head scatter with MLC.[1–4] The leaf setting strategy and effect of leaf width on physical dose distributions were discussed by Yu *et al*. and Fiveash *et al*. respectively.[5,6]

Tables 1 and 2 show the comparison of PDD parameters for all the fields using the three field-defining methods for 6-MV and 18-MV photon beams. The PDD data was measured at SSD = 100 cm, for the depth ranging from 0 to 30 cm for all the fields. The measurement are performed with the three field-defining methods (i) Jaw only (MLC in park position), (ii) MLC only — (fixed jaws opening at 35×35 cm), and (iii) MLC+Jaw (jaw optimized to MLC irregular ‘MLC+Jaw’ Fields.). From Tables 1 and 2, it is clear that the surface dose (S-0) for 6 MV and 18 MV was higher for field sizes defined by ‘MLC only’ than that for field sizes defined by ‘Jaw only’ and ‘MLC+Jaw’. The ‘percentage of difference’ of surface dose between ‘MLC only’ and ‘MLC+Jaw’ fields was (2% to 3%) and (3.5% to 5%) for 6-MV and 18-MV photons respectively. It was found that the dose in the build-up region was higher for the ‘MLC only’ field than that for the other two field-defining methods in both energies. It was found that the position of ‘depth of dose maximum’ (dmax) shifted more towards the surface for 18 MV compared to 6 MV, with increase in the field size. No significant difference in the PDD was observed beyond the dmax, that is, depth between dmax to 30 cm, for both the energies.[1] The difference in the ‘depth of 80% dose’ was within 0.5 to 1.5 mm for 6-MV and 1 to 4 mm for 18-MV photon beams for all the field defining methods. There were no significant variations when comparing the quality index (QI) with the three field-defining methods, for both the energies. The 120-leaf MLC system, which has a 5 mm leaf width, improves the dose coverage to the tumor volume and is useful in organ avoidance in head and neck tumors.[6] This will produce an ideal DVH curve for a tumor volume which gives uniform dose distribution; and at the same time, DVH of critical organs will show minimum dose contribution. In studies,[7–13] various dosimetric characteristics of multileaf collimators are measured and analyzed. The increase in the surface dose and dose rate will definitely not have any effect on the dose to the tumor, and there shall be no consequence on the clinical approach and outcome.

**Table 1:** Comparison of percentage depth dose parameters for 6-MV photon beam

*Field size (cm×cm)*	*Field defining method*	*Percentage depth dose (%)*				*Depth of 80% dose (mm)*	*Quality index*

		*Dmax*	*D-0*	*D-100*	*D-200*		*(Ql)*
	Jaw only	16	46.97	63.29	34.81	61.22	0.6344
5×5	MLC only	16	46.98	63.39	34.66	60.12	0.6302
	Jaw+MLC	16	44.41	63.68	35	60.97	0.6341
	Jaw only	16	50.84	67.1	38.52	65.86	0.6664
10×10	MLC only	16	51.37	67.04	38.62	65.65	0.6693
	Jaw+MLC	18	48.94	66.94	38.5	66.19	0.668
	Jaw only	16	59.02	70.3	42.76	71.67	0.7115
20×20	MLC only	16	60.41	69.65	42.82	69.37	0.7201
	Jaw+MLC	17	57.33	69.95	42.85	72.1	0.7172
	Jaw only	14	65.4	71.05	44.8	73.08	0.7402
30×30	MLC only	12	66.68	71.32	44.72	74.68	0.7357
	Jaw+MLC	16	63.8	71.26	44.78	73.7	0.7374

**Table 2:** Comparison of percentage depth dose parameters for 18-MV photon beam

*Field size (cm×cm)*	*Field defining method*	*Percentage depth dose (%)*				*Depth of 80% dose (mm)*	*Quality index*

		*Dmax*	*D-0*	*D-100*	*D-200*		*(Ql)*
	Jaw only	35	24.1	79.23	51.27	97.87	0.7611
(5×5)	MLC only	41	27.47	79.04	50.78	97.18	0.7553
	Jaw+MLC	37.5	22.43	79.8	51.53	99.3	0.7595
	Jaw only	33	33.43	79.27	52.73	97.92	0.7835
10×10	MLC only	30	36.9	78.25	51.37	94.7	0.7728
	Jaw+MLC	30	31.59	79.52	52.74	98.66	0.7811
	Jaw only	30	45.68	78.41	53.66	95.21	0.8068
20×20	MLC only	24	50.21	77.25	52.86	90.85	0.8066
	Jaw+MLC	25.5	45.31	78.15	53.43	94.09	0.8059
	Jaw only	27	52.64	78.7	54.46	95.73	0.8159
30×30	MLC only	21	57.1	77.84	53.43	92.81	0.8092
	Jaw+MLC	22.5	53.55	77.8	54.01	93.66	0.8184

## Beam profiles

**Figure 3:**
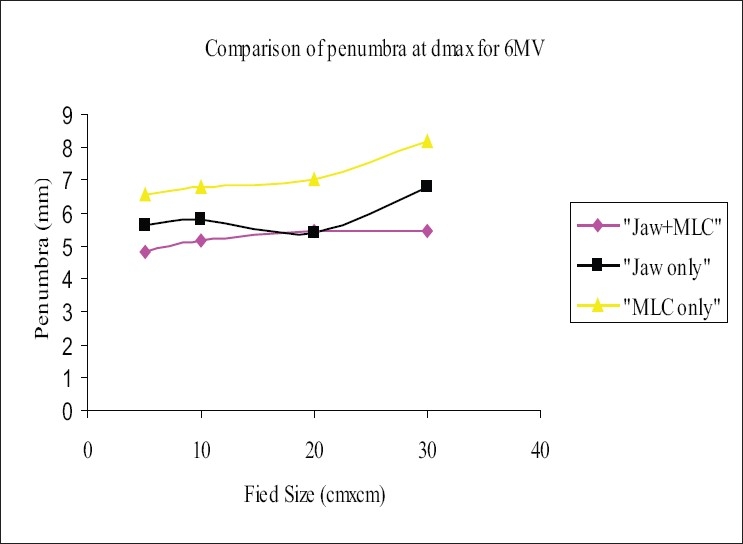
Comparison of penumbra at dmax for 6-MV photon beam, ‘Jaw only’ uses standard collimator jaws for field definition, ‘MLC only’ MLC field with jaws parked at 35×35 cm, non-optimization method, ‘MLC+Jaw’ MLC field with jaws optimized (zero gap), optimization method

**Figure 4:**
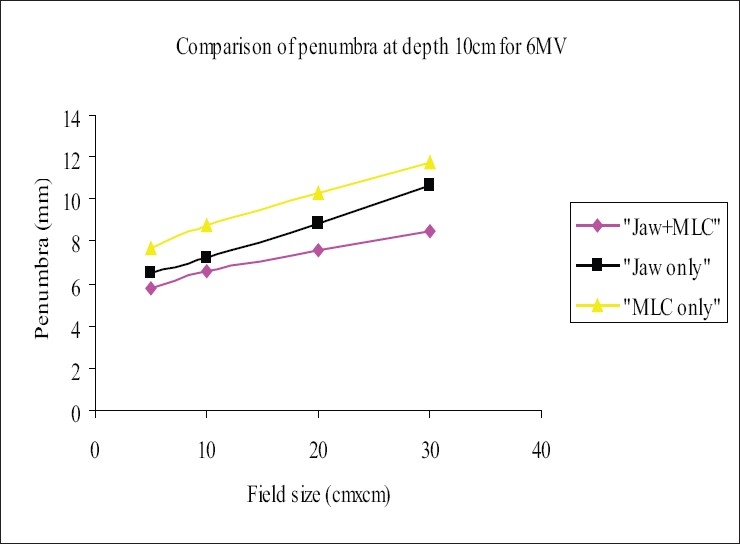
Comparison of penumbra at depth 10 cm for 6-MV photon beam, ‘Jaw only’ uses standard collimator jaws for field definition, ‘MLC only’ MLC field with jaws parked at 35×35 cm, non-optimization method, ‘MLC+Jaw’ MLC field with jaws optimized (zero gap), optimization method

**Figure 5:**
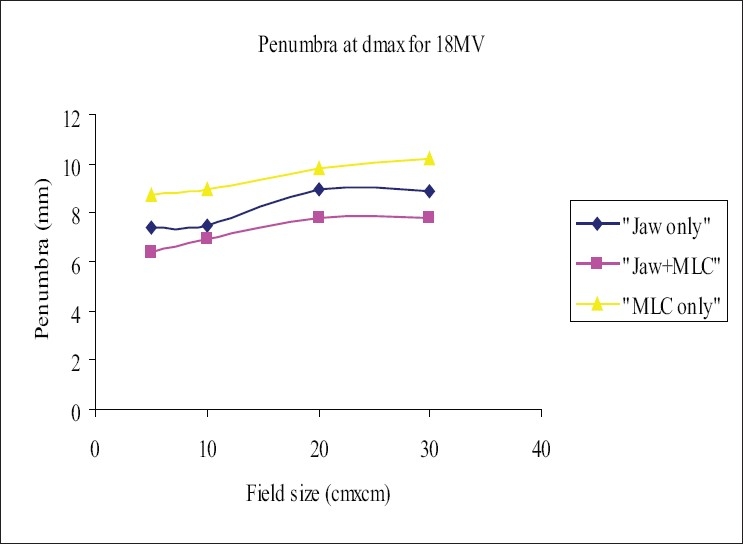
Comparison of penumbra at dmax for 18-MV photon beam, ‘Jaw only’ uses standard collimator jaws for field definition, ‘MLC only’ MLC field with jaws parked at 35×35 cm, non-optimization method, ‘MLC+Jaw’ MLC field with jaws optimized (zero gap), optimization method

**Figure 6:**
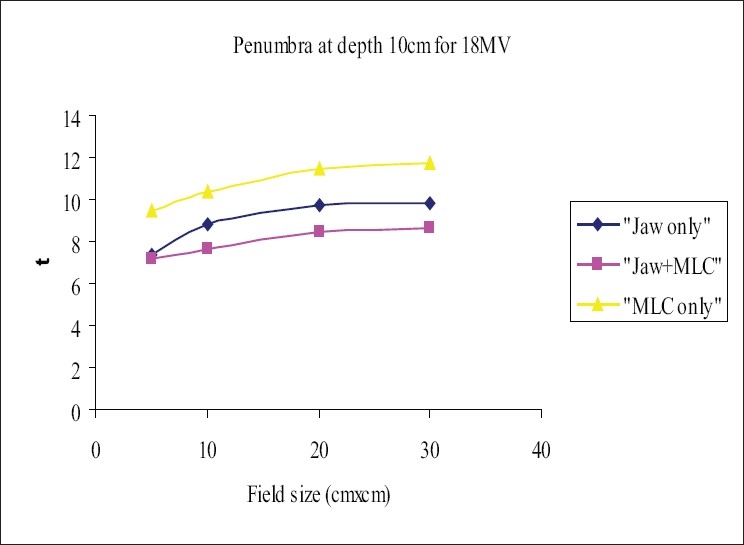
Comparison of penumbra at depth 10 cm for 18-MV photon beam, ‘Jaw only’ uses standard collimator jaws for field definition, ‘MLC only’ MLC field with jaws parked at 35×35 cm, non-optimization method, ‘MLC+Jaw’ MLC field with jaws optimized (zero gap), optimization method

The beam profiles were measured using 120-leaf MLC system, for the square field sizes 5×5 cm², 10×10 cm², 20×20 cm², and 30×30 cm² at dmax and 10 cm in the cross-plane orientation for the three field-defining methods mentioned in section D. The flatness and symmetry of the beam profiles were determined for the fields defined above; it was found that the flatness and symmetry were within 3% for both energies. The ‘width of 50% dose level’ was measured and analyzed; it was observed that the width of the ‘MLC only’ field was higher by 2 to 4 mm for 6-MV and 1 to 2.8 mm for 18-MV photon when compared with ‘Jaw only’ and/or ‘MLC+ Jaw’ fields. Tables 3 and 4 show the comparison of the ‘widths of the 50% dose level’ in the three field-defining methods for 6 MV and 18 MV respectively. The penumbra (80% to 20%) was measured at depth dmax and 10 cm. Penumbra of ‘MLC only’ field was more than that of ‘Jaw only’ and ‘MLC+ Jaw’ by 1.5 to 3 mm for 6-MV and 2 to 3 mm for 18-MV photon beam. Figures 3 and 5 show the penumbra at depth dmax for 6 MV and 18 MV respectively, and Figures 4 and 6 show the penumbra at depth 10 cm for 6 MV and 18 MV respectively. A study[14] compared the penumbra width (80% to 20%) of 10 mm leaf of three manufacturers and found that the smallest was in the Varian MLC system.

**Table 3:** Width of 50% dose level for 6 MV

*Field width at SSD = 100cm (mm)*	*Width of 50% dose level defined by ”MLC+Jaw” field*	*Width of 50% dose level defined by ”Jaw only” field (mm)*	*Width of 50% dose level defined by ”MLC only” field (mm)*
50	50.3	51.1	52.7
100	101.1	102.1	103.3
200	202.4	203.9	204.6
300	304	307.2	308.3

**Table 4:** Width of 50% dose level for 18 MV

*Field width at SSD = 100cm (mm)*	*Width of 50% dose level defined by ”MLC+Jaw” field*	*Width of 50% dose level defined by ”Jaw only” field (mm)*	*Width of 50% dose level defined by ”MLC only” field (mm)*
50	50.9	51.5	53.7
100	102.8	102.5	105.2
200	206.3	207.2	208.4
300	308.3	309.2	311.1

## Conclusion

The dosimetric characteristics of the Varian 120-leaf MLC system were measured, compared, and analyzed using 6-MV and 18-MV photon beams. It was found that its characteristics were quite similar to those of the standard collimator (jaws) system except for the dose rate, surface dose, dose in the build-up region, width of 50% dose level and penumbra. Dose rate for 6-MV and 18-MV photon beams was higher for ‘MLC only’ field than that for the other two field-defining methods. The PDD comparison shows that the surface dose and dose in the build-up region were more for ‘MLC only’ fields. Beam profile analysis shows that the flatness and symmetry for both the systems were within 3%; the ‘width of 50% dose level’ and penumbra were slightly higher for ‘MLC only’ fields in both energies. The 120-leaf MLC system with 5 mm leaf width showed improved dose coverage to the tumor volume and was found to be useful in organ avoidance in head and neck tumors. The results of this study suggest that standard collimator jaws should be optimized to the irregular MLC field (i.e., MLC+Jaw) to minimize the surface dose, dose rate, penumbra, and dose in the build-up region.
